# Void structure stability in wet granular matter and its application to crab burrows and cometary pits

**DOI:** 10.1038/s41598-018-33978-8

**Published:** 2018-10-25

**Authors:** Ayuko Shinoda, Shin-ichi Fujiwara, Hirofumi Niiya, Hiroaki Katsuragi

**Affiliations:** 1 Department of Earth and Environmental Sciences, Nagoya University, Nagoya, 464-8601 Japan; 2 Museum, Nagoya University, Nagoya, 464-8601 Japan; 3 Center for Transdisciplinary Research, Niigata University, Niigata, 950-2181 Japan

## Abstract

Cohesive granular matter can support stable void structures, which can universally be found in various scenes from everyday lives to space. To quantitatively characterize the stability and strength of a void structure in cohesive granular matter, we perform a simple tunnel-compression experiment with wet granular matter. In the experiment, a horizontal tunnel in a wet granular layer is vertically compressed with a slow compression rate. The experimental result suggests that the tunnel deformation can be classified into the following three types: (i) shrink, (ii) shrink with collapse, and (iii) subsidence by collapse. Using the experimental result, we estimate the stable limit of various void structures in a cohesive granular layer from crab burrows on a sandy beach to the pits observed on cometary surfaces.

## Introduction

On the earth, various types of natural and artificial void structures can be found in soil. Many tunnels have been constructed through mountains or under the ground to develop transportation facilities. In addition, some animals burrow into the soil to dwell in and to escape from predators. A tunnel in a sand castle constructed during children’s sand play is also a type of void in soil-like material. Even in space, void structures might be ubiquitous. For example, intact lava tubes have recently been found on the Moon^1^. Moreover, pit structures observed on the surface of comets might be caused by the collapse of internal voids^2^. The mechanical properties of soils supporting these void structures are key quantities to discuss their persistence. To estimate the collapse condition, we have to understand the mechanical stability, strength, and deformation mode of tunnels. In civil engineering, the stability of tunnel structures has been studied^3–7^. In these studies, the stability of tunnel structures even in the excavation process has mainly been studied. In general, tunnel wall reinforcement is necessary to maintain a stable tunnel in soft soil.

Some tidal and shore animals dig unreinforced burrows without any knowledge of civil engineering. Among these, ghost crabs (*Ocypode*) are known to dig deep I-, J-, or Y-shaped burrows (up to ~1 m in depth) in sandy substrates^8^. In fact, previous observations revealed that unreinforced burrows can exist in unconsolidated sandy substrates^8,9^. Particularly, Seike and Nara reported that the moisture sufficiently strengthens the burrows to retain their structures^8^. However, any quantitative characterization of burrow structure strength has not been investigated.

A crab burrow in sandy shore can be regarded as a type of void structure in wet granular matter. Thus, its stability relates to the mechanical properties of wet granular matter, which usually exhibits complex behaviours^10–14^. Such complex behaviours of wet granular matter basically stem from the variation of the liquid structural morphology within the wet granular matter^13,14^. Although some studies exist on the mechanical characterization of wet granular matter^14–23^, little is known about the stability and/or strength of void structures formed in wet granular layers. For instance, while strength of a vertical beam of wet granular matter has been estimated^22^, the stability of a void in wet granular matter has not been investigated thus far. Therefore, in this study, we perform a simple void-compression experiment with wet granular matter. Using the experimental result, we discuss burrow stability on a sandy beach. In civil engineering, centrifugal acceleration has been used to mimic the behaviour of large-scale tunnels by small-scale laboratory experiments^3,4^. For centimetre-scale crab burrows, however, direct laboratory experiments are sufficient to directly study their mechanical properties.

The void structure in cohesive granular matter could also relate to surface terrains on comets. Since comets consist of a mixture of solid grains and ice, their surface dynamics are governed by cohesive granular mechanics. Although liquid water is basically absent in space, wet granular matter is the simplest instance of cohesive granular matter. By the detailed observation of comet surfaces, various pits have been found^2,24^. Although the origin of these pits is still controversial, a recent study of comet 67 P/Churyumov-Gerasimenko^2^ suggests that pit structures result from the collapse of voids due to the sublimation of volatile materials in the comet. The physical mechanism governing the cometary pit size may be better understood based on our experimental results.

**Figure 1:**
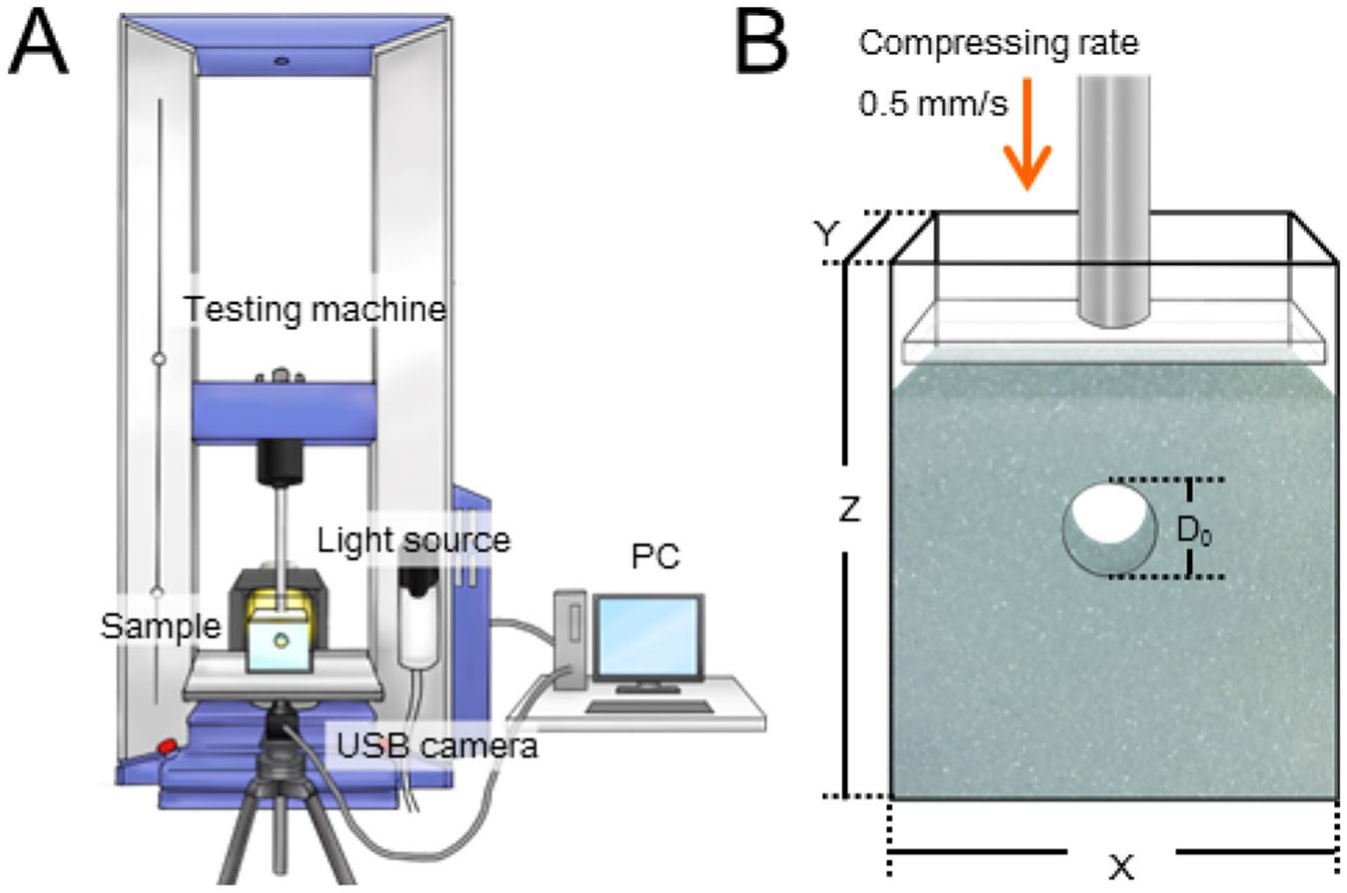
(**A**) Experimental setup including the loading machine. (**B**) Magnified view of a sample of a wet granular layer with a horizontal tunnel. On the walls of the acrylic container, holes of diameter *D*_0_ are opened on both sides. *X*, *Y*, and *Z* denote the dimensions as shown in (**B**).

In the experiment, a wet granular layer with a horizontal circular tunnel is vertically compressed by a piston with a constant speed (0.5 mm s^−1^), which is sufficiently slow to neglect the inertial effect (Fig. 1). During compression, the tunnel deformation and loading force are recorded. Using these data, we characterize the mechanical property of a tunnel structure in wet granular matter. The control parameters in this experiment are the initial tunnel diameter 5 ≤ *D*_0_ ≤ 80 (mm), grain diameter 0.1 ≤ *d* ≤ 0.8 (mm), initial volumetric liquid content 0.013 ≤ *W*_0_ ≤ 0.33, and initial solid fraction (packing fraction) $0.44\lesssim {\varphi }_{0}\lesssim 0.56$. Spherical glass beads are used in the experiment. The details of the experimental protocol can be found in the Methods section.

## Results and Analyses

### Deformation mode of a loaded void

**Figure 2:**
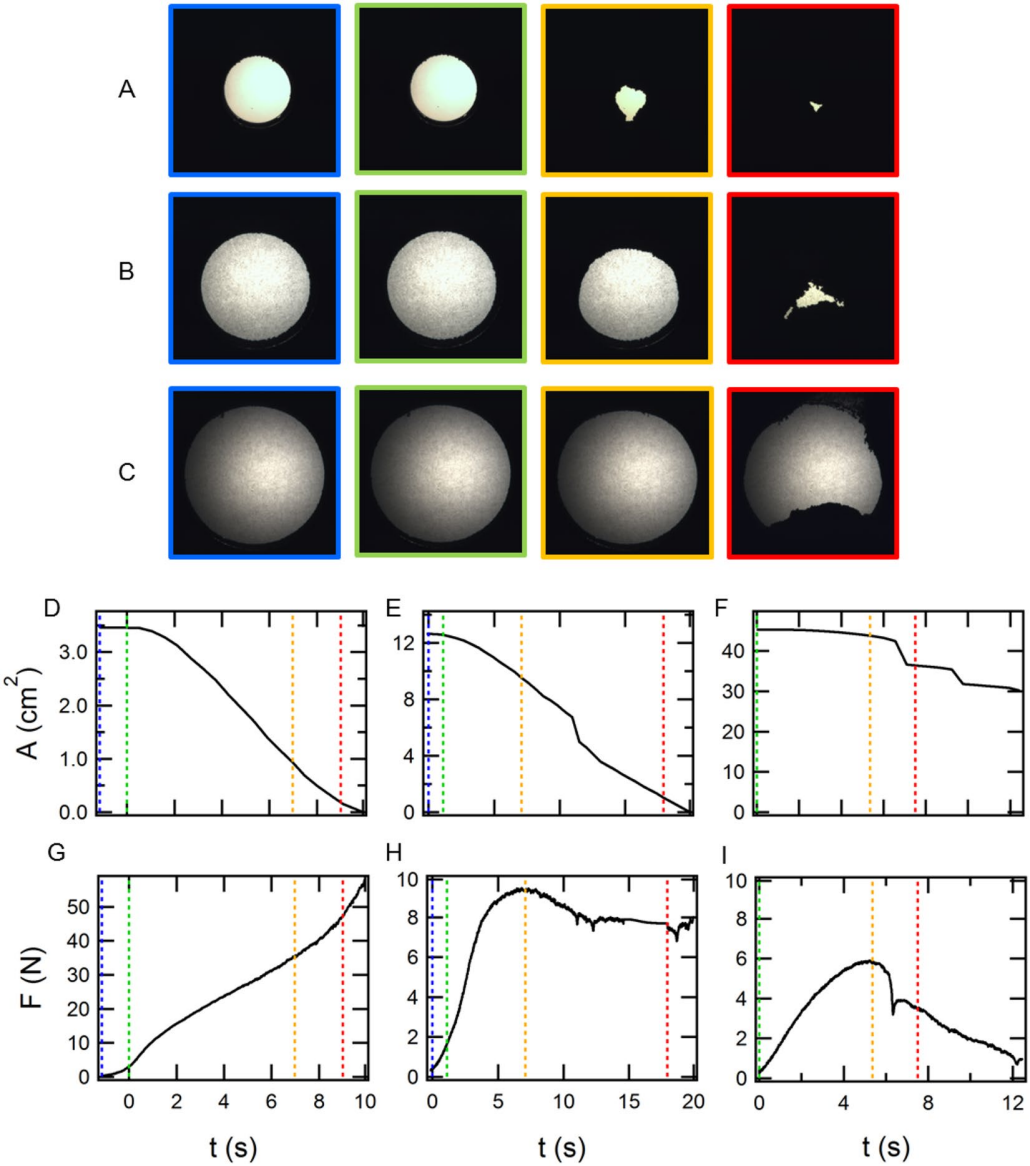
(**A**–**C**) Example images of tunnel deformation (*W*_0_ = 0.028, *ϕ*_0_ = 0.55, and *d* = 0.4 mm), temporal evolution of (**D**–**F**) cross-section *A*, and (**G**–**I**) loading force *F*. We classify the tunnel-deformation mode into three types: (**A**,**D**,**G**) shrink (*D*_0_ = 21 mm), (**B**,**E**,**H**) shrink with collapse (*D*_0_ = 40 mm), and (**C**,**F**,**I**) subsidence by collapse (*D*_0_ = 75 mm). The colour of the vertical dotted lines in (**D**,**G**), (**E**,**H**), and (**F**,**I**) corresponds to frame colours in (**A**), (**B**), and (**C**), respectively.

Typical images of compressed tunnel structures are shown in Fig. 2(A–C). By the systematic experiments, we find that the deformation mode can be classified into the following three types: (i) shrink (Fig. 2(A)), (ii) shrink with collapse (Fig. 2(B)), and (iii) subsidence by collapse (Fig. 2(C)). In the case of (i), a tunnel gradually shrinks without collapse. In type (ii), part of the tunnel ceiling suddenly collapses during its shrink. In type (iii), the ceiling of the tunnel completely falls down, causing a catastrophic subsidence.

From the acquired tunnel images, the area of the cross-section of the tunnel *A* and the corresponding diameter *D* (=2(*A*/*π*)^1/2^) are calculated. In Fig. 2(D–F), temporal evolution of *A* is displayed. The loading force *F* required to compress the layer is presented in Fig. 2(G–I). In Fig. 2, the colour of the vertical dotted lines in each plot corresponds to that of the outline of the tunnel-deformation images. Since the granular layer is compressed at a constant compression rate, *A* monotonically decreases over time. Sharp decreases in *A*(*t*) indicate void collapse. When a tunnel shrinks without collapse, *F* monotonously increases. In contrast, *F* shows a certain maximum in the collapse cases similar to plastic deformation. Perhaps, these deformation modes might not be intuitively very surprising. Nevertheless, to the best of our knowledge, such a deformation mode classification of the compressed void in wet granular matter has not been reported in neither civil engineering nor granular physics fields.

**Figure 3:**
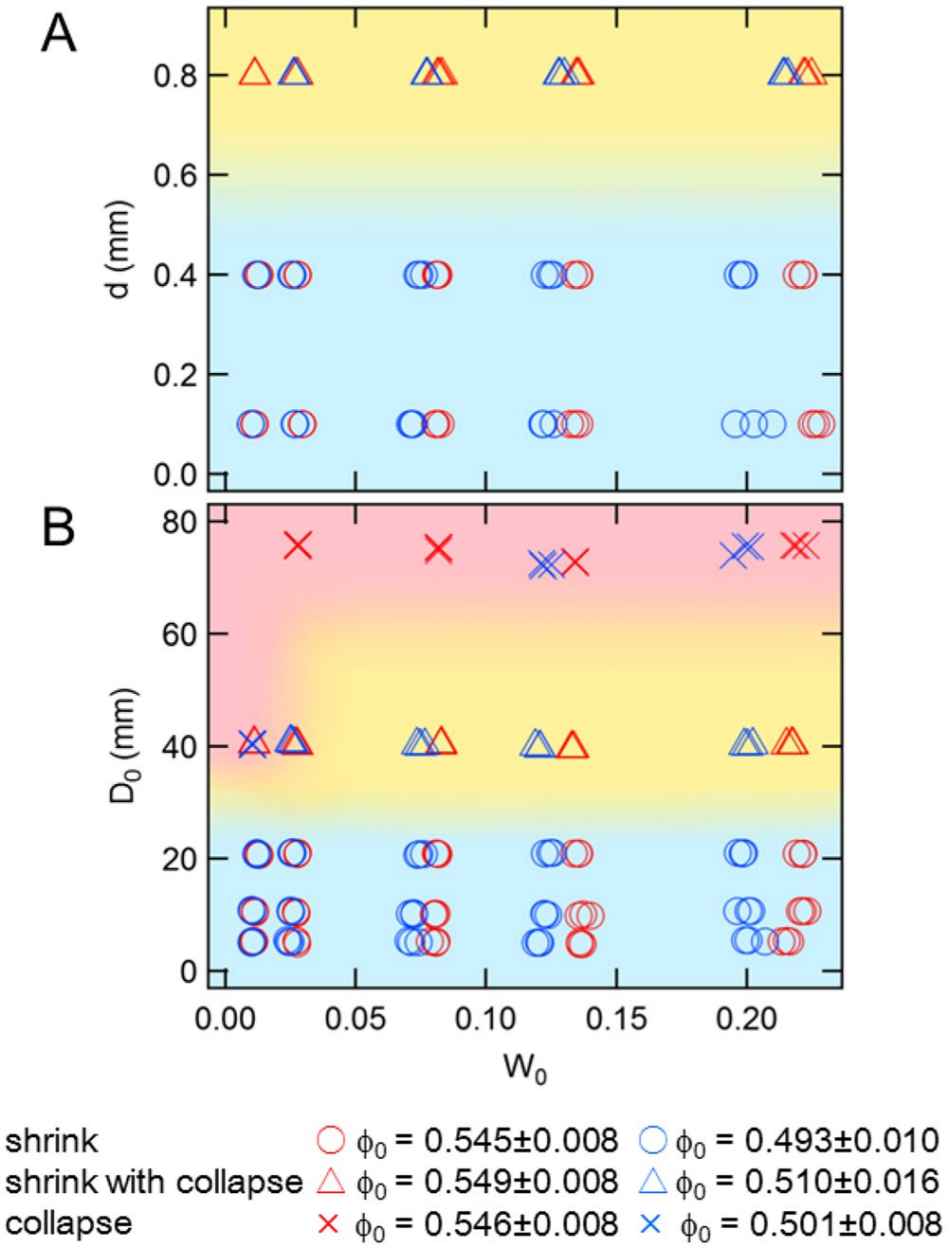
Phase diagrams of deformation mode. In (**A**), grain size *d* and liquid content *W*_0_ are varied under a constant initial tunnel diameter *D*_0_ ≃ 20 mm. In (**B**), *D*_0_ and *W*_0_ are varied under a constant *d* = 0.4 mm condition. The symbols indicate the mode of deformation, and the colour of the symbols distinguishes the initial packing fraction *ϕ*_0_.

In order to investigate which parameter significantly affects the tunnel-deformation mode, we make phase diagrams of the deformation mode as shown in Fig. 3. From these phase diagrams, one can confirm that the deformation mode is mainly dependent on *d* and *D*_0_ but almost independent of *W*_0_ and *ϕ*_0_. The large *d* or *D*_0_ results in sudden collapse. Note that the deformation mode is determined by the initial diameter *D*_0_ rather than the instantaneous diameter *D*. Additionally, the dimensionless factor *D*_0_/*d* cannot unify the phase diagrams. Both *d* and *D*_0_ must be sufficiently small to stabilize the tunnel structure. Thus, we have to consider the individual mechanisms for the instability due to large *d* or *D*_0_. We consider these effects as follows. The *d*-dependent weakening of wet granular matter is not surprising. Because the larger *d* yields the larger radius of curvature of capillary bridges among grains, it weakens the cohesive effect due to the decrease of Laplace pressure. However, *D*_0_-dependent weakening of tunnel structure cannot be easily understood. The simplest way to characterize the *D*_0_ dependence could be a balance between a mass-defect-based stress and material strength. Here, the mass-defect-based stress Δ*σ*_0_ can be estimated as Δ*σ*_0_ = *ρgV*_void_/*S*_proj_, where *ρ*, *g*, *V*_void_, and *S*_porj_ correspond to the bulk density of the wet granular layer, gravitational acceleration, void volume, and area of the void projected to a horizontal (perpendicular to gravity) surface, respectively. If Δ*σ*_0_, which roughly corresponds to the defect of static soil pressure (${\rm{\Delta }}{\sigma }_{0}\, \sim \,\rho g{D}_{0}$), exceeds the material strength, the void structure becomes unstable causing the collapse even with very small additional loading. Namely, Δ*σ*_0_ effectively corresponds to the initial loading stress due to void existence. If there is not any void structure, the initial loading is absent; Δ*σ*_0_ = 0. When Δ*σ*_0_ exceeds the material strength, the structure becomes unstable, leading to collapse. To evaluate this condition, we have to estimate the effective material strength of a void in wet granular matter.

### Strength of tunnel structures

**Figure 4:**
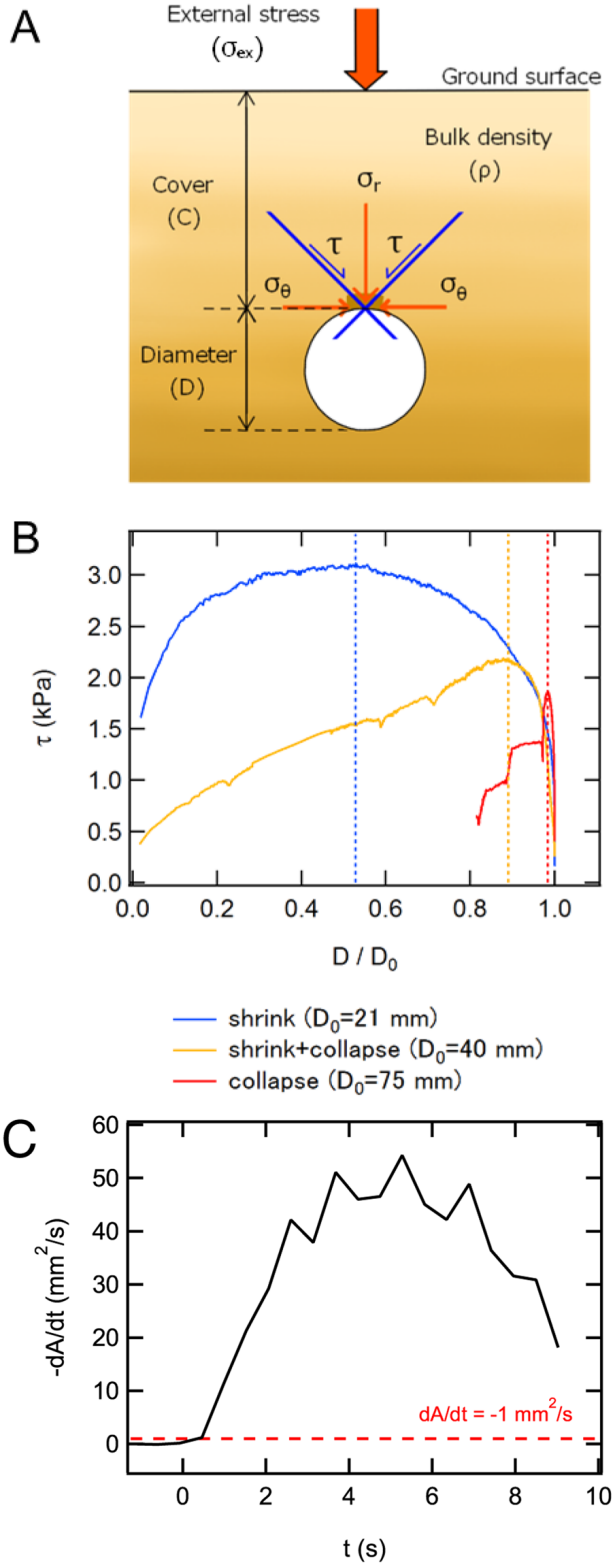
(**A**) Schematic of the stress model for a two-dimensional tunnel^25^. (**B**) The maximum shear stress *τ* as a function of the normalized tunnel diameter *D*/*D*_0_. Note that the initial state corresponds to *D*/*D*_0_ = 1, and the compression proceeds from right (*D*/*D*_0_ = 1) to left (*D*/*D*_0_ = 0). The experimental data of shrink (blue), shrink with collapse (yellow), and subsidence by collapse (red) correspond to those in Fig. 2(A–C), respectively. (**C**) An example of temporal development of *dA*/*dt* and the threshold of the yielding |*dA*/*dt*| = 1 mm^2^ s^−1^.

To estimate the tunnel-based strength, we employ a simple model for the maximum shear stress applied to a tunnel structure in soil^25^. In this model, the maximum shear stress acting on the tunnel structure, *τ*, is obtained from a static force balance in the vertical direction as^25^

1 $$\tau =\frac{{\sigma }_{{\rm{ex}}}+\rho gC}{2\,\mathrm{ln}\,\mathrm{(2}C/D+\mathrm{1)}},$$

where *σ*_ex_ = *F*/*XY* is the stress computed from the external loading force *F* acting on the top surface area *XY* (Fig. 1). *C* is thickness of cover upon a tunnel (Fig. 4(A)). As shown in Fig. 4(A), the two-dimensional cross-section of the tunnel is considered in the model, and *τ* corresponds to the shear stress acting on the plane of 45° tilted from the vertical direction. In this model, we simply consider the magnitude of stress *τ* at the top part of tunnel. We assume that when *τ* exceeds the material strength, deformation or fracturing is induced. Figure 4(B) shows *τ* computed using Eq. (1) as a function of *D*/*D*_0_. The tunnel diameter *D* is measured from the acquired images. The bulk density *ρ* is computed from the volume of the compressed wet granular layer. Other parameters are set by initial condition or constant. At the beginning of loading (*D*/*D*_0_ ≃ 1), *τ* increases rapidly. When *D*_0_ is small (21 mm), continuous shrinking of the tunnel occurs and *τ* reaches ≃ 3 kPa. In the subsidence case (*D*_0_ = 75 mm), however, *τ* shows a sharp peak due to the collapse without significant shrink. The maximum value of *τ* in this case is less than 2 kPa. The shrink-and-collapse phase (*D*_0_ = 40 mm) shows intermediate behaviour between them. To estimate the stability limit, we define the characteristic strength by the stress value at which the tunnel deformation begins, *τ*_yield_. We define the onset of deformation by the threshold of the diminution rate, |*dA*/*dt*| ≥ 1 mm^2^ s^−1^. An example of *dA*/*dt* is shown in Fig. 4(C) in which the sudden contraction of *A* can clearly be confirmed above the threshold level. Since most of the *dA*/*dt* data show a similar trend, we choose this specific threshold value. However, the *τ*_yield_ value depends on the threshold value. This arbitrariness actually results in the large variance of *τ*_yield_ as discussed later. In addition, we define another strength of the tunnel structure *τ*_max_ by the peak of *τ*. Peaks in stress curves (Fig. 4(B)) for the shrink-with-collapse and subsidence-by-collapse phases come from the actual failure of the tunnel shape, as shown in Fig. 2(H,I). However, the shrink phase does not show any sudden collapse and corresponding decrease in *F* (Fig. 2(G)). The peak of the stress curve for the shrink-phase tunnel shown in Fig. 4(B) comes from the decrease in *D*. In Eq. (1), the smaller *D* results in smaller *τ*. When this effect overcomes the effect of *σ*_ex_ increase, *τ* decreases. In other words, to keep *τ* sufficiently small (below the strength level), the tunnel shrinks in the shrink phase. Therefore, the maximum of *τ* in the shrink phase can also represent the bulk strength of wet granular matter. In the following analysis, we mainly focus on the shrink-phase tunnels to estimate the strengths using Eq. (1).

**Figure 5:**
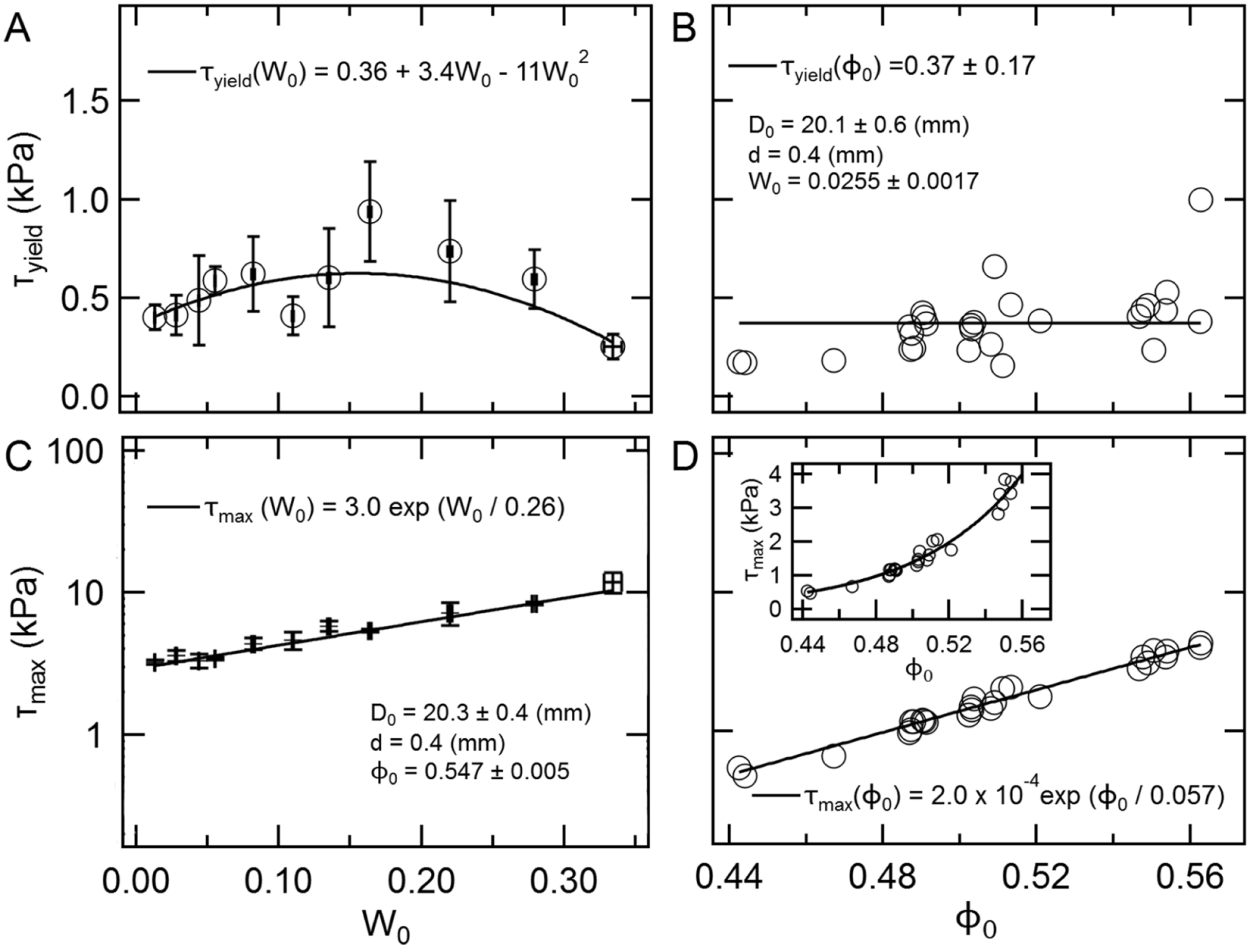
(**A**) *W*_0_ dependence of *τ*_yield_ (*ϕ*_0_ = 0.547 ± 0.005 and *D*_0_ = 20.3 ± 0.4 mm). The mean values of three runs with the identical experimental conditions are shown, and the error bars represent standard error. The curve is the fit, ${\tau }_{{\rm{yield}}}({W}_{0})={\tau }_{0}+{\tau }_{1}{W}_{0}+{\tau }_{3}{W}_{0}^{2}$, where *τ*_0_ = 0.36, *τ*_1_ = 3.4, and *τ*_2_ = −11 (kPa) are the values obtained by the fit. (**B**) *ϕ*_0_ dependence of *τ*_yield_ (*W*_0_ = 0.0255 ± 0.0017 and *D*_0_ = 20.1 ± 0.6 mm). The horizontal line represents the mean value of all plots. (**C**) *W*_0_ dependence of *τ*_max_ (*ϕ*_0_ = 0.547 ± 0.005, *D*_0_ = 20.3 ± 0.4 mm). The mean values of three runs are shown, and the error bars represent standard error. The line is the fit, *τ*_max_(*W*_0_) = *τ*_0_ exp (*W*_0_/W^*^), where *τ*_0_ = 3.0 kPa and W^*^ = 0.26 are the values obtained by the fit. (**D**) *ϕ*_0_ dependence of *τ*_max_ (*W*_0_ = 0.0255 ± 0.0017, *D*_0_ = 20.1 ± 0.6 mm). The line is the fit, *τ*_max_(*ϕ*_0_) = *τ*_0_ exp (*ϕ*_0_/ϕ^*^), where *τ*_0_ = 2.0 × 10^−4^ kPa and ϕ^*^ = 0.057 are the values obtained by the fit. The inset shows the same data in linear style.

The dependencies of *τ*_yield_ on *W*_0_ and *ϕ*_0_ are shown in Fig. 5(A,B), respectively. *τ*_yield_ distributes around 0.5 kPa. The order of these experimentally obtained strength values (~10^0^ kPa) is consistent with previous studies on strength of wet granular matter^11^. *τ*_yield_ increases with *W*_0_ at small *W*_0_ regime and shows a peak around *W*_0_ = 0.15. Liquid bridges among grains strengthens the granular layer in *W*_0_ ≤ 0.15. Then, *τ*_yield_ decreases at a larger *W*_0_ regime because liquid bridges are replaced by liquid clusters, which provide a lubrication effect^20^. However, *W*_0_ dependence of *τ*_yield_ is very weak; thus, we can assume it is approximately a constant. *τ*_yield_ does not clearly depend on *ϕ*_0_. The large variance of *τ*_yield_ vs. *ϕ*_0_ (Fig. 5 (B)) could originate from the effect of the fixed threshold for computing *τ*_yield_. Although the value of *τ*_yield_ depends on the threshold of *dA*/*dt*, the qualitative behaviour and the order of magnitude of *τ*_yield_ are almost independent of the threshold value.

To evaluate the instability of the void structure, the comparison between Δ*σ*_0_ and these strength values would be helpful. By substituting typical values *ρ *= 1.4 × 10^3^ kg m^−3^ and *V*_void_/*S*_proj_ = *πD*_0_/4 with the typical initial diameter of collapsing tunnel *D*_0_ = 50 mm into the form of Δ*σ*_0_, we obtain ${\rm{\Delta }}{\sigma }_{0}\simeq 0.54$ kPa. This value is actually close to *τ*_yield_ ≃ 0.5 kPa. That is, when a dimensionless number *χ* = Δ*σ*_0_/*τ*_yield_ exceeds unity, the initial void structure is unstable and subsidence by collapse could be induced by small amount of additional loading. Since *τ*_yield_ is relatively independent of *W*_0_, *D*_0_ is the principal variable in *χ*. That is the reason why the phase boundaries in Fig. 3 are almost horizontal. When *χ* is sufficiently less than unity, on the other hand, continuous shrink is induced by the compression.

Figure 5 (C,D) show the variations of *τ*_max_ as a function of *W*_0_ and *ϕ*_0_. The value of *τ*_max_ is approximately in the range of 1–10 kPa. *τ*_max_ monotonically increases with *W*_0_. This trend differs from that of *τ*_yield_ because the effect of compaction is not negligible for *τ*_max_. When the granular layer is loaded, pressed grains contribute to not only filling a tunnel hole but also increasing packing fraction of the matrix (bulk) layer. Since the wetting liquid allows for grains to move smoothly, the granular layer can be easily compacted. Whereas this matrix-compression effect is limited in the early stage of deformation, it plays a certain role in the late stage determining *τ*_max_. As shown in Fig. 5(D), *τ*_max_ increases with *ϕ*_0_. This trend likely originates from the increase of contact points among grains where new liquid bridges can be formed. Some empirical fitting forms are also presented in Fig. 5 only to guide the eye. Since the variations of *D*_0_ and *d* are limited in this experiment, *D*_0_ and *d* dependencies of *τ*_yield_ and *τ*_max_ can only be roughly discussed. While the data are not shown here, we find that both *τ*_yield_ and *τ*_max_ are almost independent of *D*_0_ and are decreasing functions of *d*. Details of the systematic analyses of the entire data set and the empirical forms for estimating strength values are found in^26^.

## Discussion

Using the experimental result, the upper limit of crab burrow size can be discussed. We have also investigated crab burrows at a sandy beach in Tsu, Mie prefecture, Japan. The average diameter of the actual crab burrows we found is 26.4 mm, and the standard deviation is 6.2 mm. In addition, the water content *W* of the region at which crab burrows exist is in the range of 0.1 ≤ *W* ≤ 0.4. These observations are consistent with those of previous studies^8,9^. From the observational results and phase diagrams in Fig. 3, we can conclude that burrows are safety in terms of collapse prevention. Put differently, ghost crabs make sufficiently small burrows (*D*_0_ < 50 mm) to reduce the risk of sudden collapse. Since the phase boundary does not significantly depend on *W*_0_ (at least in the range of *W* at the actual sandy beach, 0.1 ≤ *W* ≤ 0.4 (Fig. 5(A))), the water content is not very important as long as the layer is somewhat wet. This *W*_0_-insensitivity is advantageous for crabs because the water content on beach could vary depending on the weather/tide^27^.

The results presented thus far could depend on the grain shape. According to the preliminary results of the experiment with sand grains, the strength of sand qualitatively shows the similar tendency to the current experimental result obtained by glass beads. The structure of the phase diagram shown in Fig. 3 is almost identical in the sand case. A much more detailed comparison between the experimental result and actual crab burrows on the basis of field survey will be presented elsewhere.

By considering the buckling instability, the strength of a wet granular pillar was estimated^22^. According to their result, the typical strength of a wet granular pillar is on the order of MPa. This value is much greater than the strength estimated in this study. This difference is rather natural since the dense pillar structure is much stronger than the void structure. The strength obtained in this study must be used to properly discuss the stability of sand tunnel structures.

The current experimental result also enables us to discuss the size of pits observed on comet 67 P. Vincent *et al*. reported that the size of pits ranges from 50 m to 300 m^2^. In that study, they only discussed the relation between critical ceiling thickness and void diameter by assuming a simple sinkhole model. However, in their model, the size range is arbitrary. In our experiment, it turns out that the small void is stable. Moreover, the small voids result in the continuous shrink without collapse. Namely, the small voids cannot produce pits. Thus, the lower limit of size of pits could be estimated by the comparison between Δ*σ*_0_ and the strength of materials. According to Vincent *et al*.^2^, the typical values for comet 67 P are *ρ* = 470 kg m^−3^, *g* = 5 × 10^−4^ m s^−2^, and tensile strength of surface material *τ*_com_ ≃ 50 Pa. Here, we consider the collapse condition by calculating stress balance among the effective loading by the void structure Δ*σ*_0_, the yield stress *τ*_yield_, and the maximum material strength *τ*_max_ with a help of the collapse condition *χ* = Δ*σ*_0_/*τ*_yield_ ≃ 1. By assuming a spherical void shape with the lower-limit diameter of collapsing void, *D*_0_ = 50 m, Δ*σ*_0_ becomes $\rho g(\pi {D}_{0}^{3}\mathrm{/6)/(}\pi {D}_{0}^{2}\mathrm{/4)}=2\rho g{D}_{0}\mathrm{/3}\simeq 8$ Pa. To consistently explain the pit subsidence by void collapse, the effective *τ*_yield_ on the surface of a comet should roughly be identical to Δ*σ*_0_ = 8 Pa from the criterion *χ* ≃ 1. From the experimental result, the ratio between material strength *τ*_max_ and yield strength *τ*_yield_ is *τ*_max_/*τ*_yield_ ≃ 3–6 (Fig. 4(B) and *τ*_yield_ ≃ 0.5 kPa). This value is approximately consistent with the observation, *τ*_com_/*τ*_yield_ = 50/8 ≃ 6 by assuming that *τ*_max_ is governed by tensile strength. Namely, the lower limit of pit size could be determined by the stability limit of the void size in the cohesive granular layer.

## Conclusion

In summary, we conducted a simple experiment of shrink and collapse of voids in compressed wet granular matter. The phase boundary between shrink and collapse mainly depends on grain size *d* and initial void size *D*_0_. Particularly, the tunnel structure with larger *d* or *D*_0_ tends to collapse. From the experimental result, we revealed that the crab burrows are sufficiently small to prevent the subsidence hazard, and the lower-limit size of pits on comet 67 P could be governed by a similar collapse mechanism.

## Methods

In the experiment, we observe how the tunnel structure in the wet granular layer deforms when it is uniformly compressed from the top of the layer. The wet granular matter is prepared by rotating a mixture of grains and liquid (tap water, *ρ*_liquid_ = 1000 kg m^−3^) for 10 minutes with 100 rpm on the pot mill rotation table (Nittokagaku, ANZ). The tunnel structure is made by extracting a solid cylinder (of diameter *D*_0_) from the wet granular layer packed in an acrylic container with holes. Note that the solid cylinder is placed before preparing the wet granular layer and withdrawn after packing. To neglect the aging effect such as draining or evaporation of water, the granular layer is quickly prepared, and the prepared granular layer is compressed right after the preparation. We confirmed that the evaporation is sufficiently slow. Regarding draining, its effect is negligible due to the capillary force when the liquid content is not large. However, in the large liquid-content case, draining might affect the result. To minimize that effect, we quickly perform the experiment.

In this experiment, we control the following parameters: initial liquid content *W*_0_, initial solid fraction *ϕ*_0_, initial diameter of the tunnel *D*_0_, and grain size *d*. *W*_0_ is defined as the volume of liquid *V*_liquid_ divided by the total volume of the wet granular layer including solid, liquid, and void parts, *V*_total_; *W*_0_ = *V*_liquid_/*V*_total_. On the other hand, *ϕ*_0_ is defined as *ϕ*_0_ = *V*_grain_/*V*_total_, where *V*_grain_ is the volume occupied by (dry) grains. The experiments are carried out 3 times with identical initial conditions. We perform experiments over a range of *W*_0_ from 0.013 to 0.33 and *ϕ*_0_ from 0.44 to 0.56. Various size containers to vary *D*_0_ are used: (*D*_0_, X, Y, Z) = (5 mm, 75 mm, 40 mm, 100 mm), (10 mm, 80 mm, 40 mm, 105 mm), (20 mm, 100 mm, 40 mm, 150 mm), (40 mm, 110 mm, 40 mm, 135 mm), and (80 mm, 150 mm, 40 mm, 175 mm). See Fig. 1 for the definition of dimensions *X*, *Y*, and *Z*. The ceiling thickness of the granular layer above the tunnel *C* is fixed at 20.6 ± 2.0 mm at the initial state. Although there might be a certain wall effect in the experiment, we fix the depth of the cell (*Y* = 40 mm) to minimize the effect of wall effect variation. Moreover, *Y* = 40 mm is sufficiently thick to neglect the frictional wall support (so-called Janssen effect, see, e.g., Sec. 3.7 in^28^) since the layer thickness above the tunnel (*C* = 20 mm) is smaller than *Y*. Grains used in this experiment are glass beads (true density: *ρ*_grain_ = 2500 kg/m^3^, grain size: *d* = 0.1, 0.4, or 0.8 mm, AS-ONE). During the compression, movies of the cross-section of deforming tunnel is acquired by a CMOS camera (Sentech, STC-MCCM401U3V (4 M Color)) at a rate of 2 frames per second by using transmitted light. We also measure the force *F* to compress the wet granular layer and displacement of the loading top plate using a universal testing machine (Shimadzu, AG-X). In this experiment, loading rate of the top plate is fixed at 0.5 mm s^−1^. We checked that this loading rate is sufficiently slow to neglect the inertial effect. The behaviour does not depend on a loading rate below the speed of 0.5 mm s^−1^. This loading rate is the fastest one in the quasi-static regime. To avoid the aging effect, the faster loading rate is better. Therefore, we employ this rate.
